# Reduced Relapse-Free Survival in Colorectal Cancer Patients with Elevated Soluble CD40 Ligand Levels Improved by Vitamin D Supplementation

**DOI:** 10.3390/nu15204361

**Published:** 2023-10-13

**Authors:** Hiroshi Fujimoto, Soichiro Fukuzato, Kazuki Kanno, Taisuke Akutsu, Hironori Ohdaira, Yutaka Suzuki, Mitsuyoshi Urashima

**Affiliations:** 1 Division of Molecular Epidemiology, The Jikei University School of Medicine, 3-25-8 Nishi-Shimbashi, Minato-ku, Tokyo 105-8461, Japan; hfujimoto519@gmail.com (H.F.); sunshine1.324g@gmail.com (S.F.); k.k.effort.patience@gmail.com (K.K.); taisuke0107.jusom@gmail.com (T.A.); 2 Biometrics and Data Sciences, Bristol-Myers Squibb K.K., 1-2-1 Otemachi Chiyoda-ku, Tokyo 100-0004, Japan; 3 Department of Surgery, International University of Health and Welfare Hospital, 537-3 Iguchi, Nasushiobara 329-2763, Japan; ohdaira@iuhw.ac.jp (H.O.); yutaka@iuhw.ac.jp (Y.S.)

**Keywords:** vitamin D, supplement, CD40 ligand, CD40L, soluble CD40 ligand, sCD40L, survival, randomized, placebo, colorectal cancer

## Abstract

Although elevated serum levels of soluble CD40 ligand (sCD40L) were reported in patients with cancer, the importance of high sCD40L levels in clinical oncology remains unknown. We conducted a post hoc analysis of the AMATERASU randomized clinical trial of vitamin D3 supplementation (2000 IU/day) in patients with digestive tract cancer to assess its significance. Serum sCD40L levels were measured by ELISA in 294 residual samples, and were divided into tertiles. In patients with colorectal cancer (CRC), 5-year relapse-free survival (RFS) rates in the middle and highest tertiles were 61.6% and 61.2%, respectively, which was significantly lower than 83.8% in the lowest tertile. A Cox proportional hazard analysis showed that the lowest tertile had a significantly lower risk of relapse or death than the highest tertile even with multivariate adjustment (hazard ratio (HR), 0.30; 95% confidence interval (CI), 0.11–0.80; *p* = 0.016). In the subgroup of CRC patients with the highest tertile of sCD40L, the 5-year RFS rate in the vitamin D group was 77.9%, which was significantly higher than 33.2% in the placebo group (HR, 0.30; 95% CI, 0.11–0.81; *p* = 0.018 [*P_interaction_ =* 0.04]). In conclusion, elevated sCD40L might be a biomarker of poor prognosis in patients with CRC, but vitamin D supplementation might improve RFS in patients with high sCD40L.

## 1. Introduction

In 2020, the global community witnessed an estimated 19.3 million new cancer cases and nearly 10.0 million cancer-related fatalities [1]. Projections indicate a sobering escalation of these figures, with expectations of 30.2 million cases and 16.3 million deaths by 2040 [2]. In response to this burgeoning crisis, a wealth of clinical evidence has been amassing, shedding light on the favorable impact of vitamin D on the survival of cancer patients [3]. To address the mounting incidence and mortality rates linked to cancer, several randomized clinical trials (RCTs) investigating vitamin D supplementation have been conducted. Among these, the VITAL trial, encompassing 25,871 participants and demonstrating the capacity of vitamin D supplements for effectively deterring the incidence of fatal cancers, stands out [4]. The study also showed a significant reduction in cancer-related deaths, a phenomenon observed two years after commencing supplementation [5]. Several meta-analyses have also shown the potential of vitamin D supplementation in curtailing overall cancer mortality [6,7,8], spanning various cancer types, including colorectal cancer (CRC) [9]. Our own investigation, the AMATERASU RCT, which enrolled patients diagnosed with stage I-III digestive tract cancers ranging from the esophagus to the rectum, revealed a 5-year relapse-free survival (RFS) rate of 77% for patients receiving vitamin D, compared to 69% for those on a placebo regimen (hazard ratio (HR) for relapse or death, 0.76; 95% confidence interval (CI), 0.50–1.14) [10]. We further scrutinized the AMATERASU trial through post hoc analyses. Notably, vitamin D supplements exhibited heightened efficacy in mitigating the risk of recurrence or mortality among patients with specific subgroups, such as elevated soluble programmed death-ligand 1 (PD-L1) levels [11].

Immunotherapy for cancer, with a particular focus on immune checkpoint inhibitors targeting PD-1 or PD-L1, has emerged as a prominent area of research and clinical interest [12]. Nevertheless, the effectiveness of PD-1/PD-L1 inhibitory immunotherapy is inconsistent, especially in patients with digestive tract cancers. This underscores the pressing need for innovative immunotherapeutic approaches. Recent in vitro experiments have unveiled the potential for dendritic cell-based immunotherapy, activated through the CD40/CD40 ligand (CD40L) immune checkpoint, to provoke robust anti-tumor responses not only in CRC [13], but also in hepatocellular carcinoma [14] and oral cancer [15]. Intriguingly, studies have demonstrated that activation of murine tumor lysate-pulsed dendritic cells by CD40L leads to a more vigorous induction of systemic immunity as compared to cells prepared without CD40L activation [16]. However, it is worth noting that an RCT investigating autologous dendritic cell vaccines, whether activated by CD40L or not, failed to yield improvements in RFS in patients with metastatic CRC [17]. Consequently, the precise significance of CD40L in the realm of immunotherapy remains unknown.

In the 1990s, the field of immunology witnessed the initial revelations surrounding CD40-CD40L interactions. The CD40 receptor was originally identified on B cells as well as B cell malignancies [18]. Moreover, CD40L was cloned and identified as a cell surface molecule expressed on activated T cells. It was found to play a pivotal role in fostering B-cell proliferation and triggering IgE production, particularly in conjunction with interleukin (IL)-4 [19]. Notably, genetic mutations in CD40L have been linked to X chromosome-linked immunodeficiency, characterized by hyper-IgM syndrome [20,21,22,23]. This firmly underscores the significance of CD40L in driving immunoglobulin class switching within B cells from IgM to IgG, IgA, and IgE, and facilitating interactions between T cells and B cells [24]. In experiments involving B cells from patients with X-linked hyper-IgM syndrome, the combination of soluble CD40 ligand (sCD40L) with IL-4 or IL-10 prompted B cell proliferation and spurred their differentiation toward the secretion of IgG, IgA, and IgE [25]. This compelling evidence suggests that sCD40L has the potential to serve as a biologically active molecule [26].

Since 1998, there has been a mounting interest in the role of sCD40L in the context of platelets, atherosclerosis, and coronary artery disease. Studies showed that activated platelets prominently express CD40L, a factor that actively contributes to the formation of thrombi [27]. Reportedly, platelet activation leads to a release of CD40L, which, in turn, triggers endothelial cells to produce chemokines and express adhesion molecules [27]. This intricate process sets the stage for recruitment and mobilization of leukocytes to the site of injury [27]. Notably, heightened levels of sCD40L were found to be indicative of an elevated risk of cardiovascular events, particularly in patients with unstable coronary artery disease [28]. Further investigations revealed that sCD40L levels were also elevated in cases of stable atherosclerosis, particularly within the carotid and coronary territories. These findings support the notion that sCD40L has the potential to serve as a marker of systemic atherosclerosis [29].

CRC is the third most frequently diagnosed cancer on a global scale, and carries the second-highest mortality rate among cancers [1]. Its development and progression are influenced by a complex interplay of factors, including gut microbiota, genetic predisposition, dietary habits, environmental influences, and, notably, inflammation [30,31]. For instance, individuals with inflammatory bowel syndrome face an elevated risk of developing CRC [32]. Within the realm of inflammatory markers, the platelet-to-lymphocyte ratio has emerged as a promising tool for evaluating the prognosis of CRC [33], indicating an association among activated platelets, elevated sCD40L, and poor prognosis of CRC patients.

Fast-forwarding to approximately 2010, researchers began delving into the relationship between sCD40L and solid cancers. Moreover, compared to healthy individuals, patients with different types of cancer, including CRC [34], lung cancer [35], and breast cancer [36], exhibit elevated serum sCD40L levels. This compelling evidence hinted at the possibility of sCD40L playing a predictive role in human carcinogenesis [37]. Specifically, an increase in plasma sCD40L levels was suggested as being closely associated with disease progression and the occurrence of metastasis in various cancer types, including gastric cancer [38], pancreatic cancer [39], nasopharyngeal carcinoma [40], and CRC [34]. These heightened sCD40L levels have been shown to have immunosuppressive effects in patients with cancer, such as promotion of regulatory T cells, up- or down-regulation of cytokine release, and enrichment of PD-1-expressing T cells [41]. Though elevated sCD40L levels are deemed indicative of the presence of a tumor [37], the significance of elevated sCD40L levels in clinical oncology remains unknown.

A recent RCT showed that vitamin D supplementation had the capacity to reduce expression of the CD40L gene in patients with ulcerative colitis [42]. This discovery opened up the prospect that vitamin D supplementation might influence RFS rates in cancer patients, particularly those presenting high levels of serum sCD40L. To investigate this hypothesis, we conducted a post hoc analysis of the AMATERASU RCT [10] and aimed to examine the influence of vitamin D supplementation on RFS within each tertile of serum sCD40L levels in patients with digestive tract cancers or CRC. In this study, in alignment with our hypothesis that vitamin D supplementation might improve RFS rates in CRC patients, we could have exclusively concentrated on CRC patients who participated in the AMATERASU trial. However, we deemed it essential to include gastric and esophageal cancer cases in our analysis for comparison.

## 2. Materials and Methods

### 2.1. Trial Design

This post hoc analysis was conducted using data from the AMATERASU trial (Trial Registration Identifier: UMIN000001977), which was performed in Japan from January 2010 to February 2018. Detailed information about the trial has been previously published [30]. Briefly, the AMATERASU trial was designed as a double-blind, placebo-controlled study comparing the effects of vitamin D3 supplements (2000 IU/day) and a placebo, with a ratio of allocation set at 3:2.

Patients who underwent surgical treatment for digestive tract cancer anywhere from the esophagus to the rectum at the International University of Health and Welfare Hospital were randomized to receive vitamin D supplementation or placebo during their first postoperative outpatient visit, which typically occurred between 2 and 4 weeks after the operation.

The trial protocol received approval from the ethics committee of the International University of Health and Welfare Hospital (Otawara, Tochigi, Japan) on 4 December 2009 (code: 13-B-263), as well as from the Jikei University School of Medicine (Nishi-shimbashi, Tokyo, Japan) on 1 January 2010 (code: 21-216 [6094]). Written informed consent was obtained from each participating patient before surgery.

### 2.2. Participants

Specifics regarding the inclusion and exclusion criteria can be found in the original report [14]. Briefly, the study included individuals aged 30–90 years at entry, with a histopathological diagnosis of clinical stage I–III epithelial carcinoma of any part of the digestive tract (including the esophagus, stomach, small intestine, colon, and rectum), who were diagnosed and initially treated at the International University of Health and Welfare Hospital.

Exclusion criteria included cases in which tumors were not resectable through surgery, instances of severe postoperative complications occurring prior to the initiation of supplementation, pathological diagnoses other than epithelial carcinoma (such as malignant lymphoma and sarcoma), pathological stage 0 or IV cancer, requirement to take vitamin D supplements or active vitamin D, and history of urinary tract stones. Collaborating surgeons discussed the trial with eligible patients and their families at outpatient clinics before the surgery and invited them to participate. All clinical data were collected at the International University of Health and Welfare Hospital, and monitored at the Division of Molecular Epidemiology of the Jikei University School of Medicine.

### 2.3. Randomization and Blinding

Randomization in this study utilized computer-generated and centrally administered methods, employing permuted blocks of 5. Participants were randomly allocated at a ratio of 3:2 without any stratification. To enhance willingness to participate, an increased likelihood of randomization to the vitamin D group was implemented.

With the exception of MU and the personnel at the data monitoring center located at the Jikei University School of Medicine, who were responsible for preparing the vitamin D or placebo bottles based on the randomized assignment, all other surgeons, the clinical research coordinator, and participants at the International University of Health and Welfare hospital were blinded to the patients’ group assignments.

### 2.4. Intervention

At their first outpatient postoperative follow-up visit, patients who had been enrolled in the study were subjected to random assignment. They were instructed to take either the vitamin D3 supplement (2000 IU/day) or placebo orally daily, beginning on the same day, until the conclusion of the trial. Both the vitamin D3 supplements and placebos were procured from Zenyaku Pharmaceutical Co., Ltd., Tokyo, Japan.

### 2.5. Follow-Up and Outcomes

As part of the study protocol, patients underwent regular outpatient examinations utilizing CT, MRI, PET scans, and other necessary procedures to monitor for any signs of cancer relapse, as determined by the attending surgeon. Typically, the examinations were conducted monthly during the initial 6 months postoperatively, bi-monthly during the subsequent 6 months, and then every 3 months for the remaining 4 years. After the initial 5 years, the follow-up frequency was adjusted to every 3–6 months, based on the patient’s condition, as assessed by the responsible surgeon.

A clinical research coordinator was actively involved in patient follow-up, conducting interviews during each outpatient clinic visit at the International University of Health and Welfare Hospital. The research coordinator contacted participants via telephone to inquire about their health status and adherence to the treatment regimen. Further, adherence to the trial supplement was reconfirmed when a new bottle was provided to the participants every six months.

In all patients in both the vitamin D and placebo groups, blood 25(OH)D levels were measured annually to monitor changes in their levels. In addition to the assigned supplementation, patients diagnosed with stage II and III esophageal, gastric, or colorectal cancer were given pre- and postoperative chemotherapy.

In select cases of relapse, local radiation or molecular targeting therapy was combined with chemotherapy. In situations where vitamin D supplementation became medically necessary, such as for conditions like bone fractures or osteoporosis, trial supplementation was discontinued. However, these patients continued to be monitored until the conclusion of the study.

The outcome of this study was prespecified as relapse or all-cause death. The elapsed time was defined as the time from starting the supplement to the date the outcome occurred or was censored.

### 2.6. Measurement of Serum sCD40L Levels

Serum samples for the measurement of sCD40L levels were obtained at a median interval of 23 days (interquartile range (IQR): 13–43.5 days) following the surgical procedures, and immediately preceding the commencement of vitamin D/placebo supplementation. These serum specimens were aliquoted in 0.5 mL portions to avoid frequent thawing and refreezing, and stored at a temperature of −80 °C until their utilization in subsequent analyses.

The quantification of serum sCD40L levels was performed by a member of the research team who conducted the measurements without any prior knowledge of the randomized group assignments or the patients’ clinical information, including their predetermined outcomes, which were established prior to the initiation of statistical analyses.

A Human CD40L ELISA Kit (TNFSF5) (ab196268) (Abcam, Cambridge, MA, USA) was used according to the manufacturer’s protocols. The lower limit of detection of serum sCD40L of the ELISA kit was 2.9 pg/mL. The 294 samples in which sCD40L was measured were divided into tertile groups based on sCD40L levels, with boundaries set at 83.35 pg/mL and 165.76 pg/mL.

### 2.7. Vitamin D Measurement

Serum 25(OH)D levels were assessed annually, using radioimmunoassay (SRL Inc., Hachioji, Tokyo, Japan), starting from the initiation of supplementation and continuing every year thereafter during the same calendar month for up to 5 years. To ensure accuracy, blinded duplicate tests for 25(OH)D were conducted using 19 serum samples from a separate cohort, demonstrating a correlation coefficient of 0.92, as previously described [43].

### 2.8. Sample Size

When analyzing raw data from a cohort of 257 CRC patients at the Jikei University School of Medicine, the 5-year RFS of the highest quartile of 25(OH)D was found to be 13% higher than that of the lowest quartile [44]. Considering approximate survival rates for digestive tract cancer in Japan, it was estimated that the 5-year RFS rates would be 75% in the vitamin D group and 62% in the placebo group, assuming a two-sided type I error of 5% and 80% power, with a 1% loss to follow-up. Estimation suggested that 400 patients with digestive tract cancers, divided in a 3:2 ratio, would be sufficient to detect this difference.

Given an expected annual enrollment of 80 patients, the accrual period was projected to be 5 years to achieve the enrollment of 400 participants. With final patient follow-up conducted 2 years later, the total duration of the planned trial was 7 years.

### 2.9. Statistical Analysis

All participants were randomized and those whose sCD40L levels in residual serum samples could be measured were included in this analysis. Categorical and continuous variables in the vitamin D and placebo groups were compared using chi-squared and Mann–Whitney tests, respectively. The serum sCD40L levels were divided into tertiles (T1–T3), and chi-square and Kruskal–Wallis tests were employed to compare categorical and continuous variables among sCD40L tertiles, considering the non-normally distributed nature of the data and the need to assess differences among more than three groups. For survival analyses, 5-year RFS rates were compared among tertiles and between vitamin D and placebo groups. Moreover, Nelson–Aalen cumulative hazard curves were created to show the changes in relapse or death risk over time. Hazard ratios (HRs) and corresponding 95% confidence intervals (CIs) were computed utilizing a Cox proportional hazard model. Interaction tests based on a Cox regression model were conducted to discern any potential divergence in the effects of vitamin D supplementation across the tertiles of serum sCD40L levels. Two-way interaction tests were employed to compare the subgroup featuring the highest tertile of serum sCD40L among CRC patients with the remaining participants. Statistical significance was indicated by two-sided *p* values < 0.05. The entirety of the analysis was executed using Stata 17.0 (StataCorp LP; College Station, TX, USA).

## 3. Results

### 3.1. Study Population

**Figure 1:**
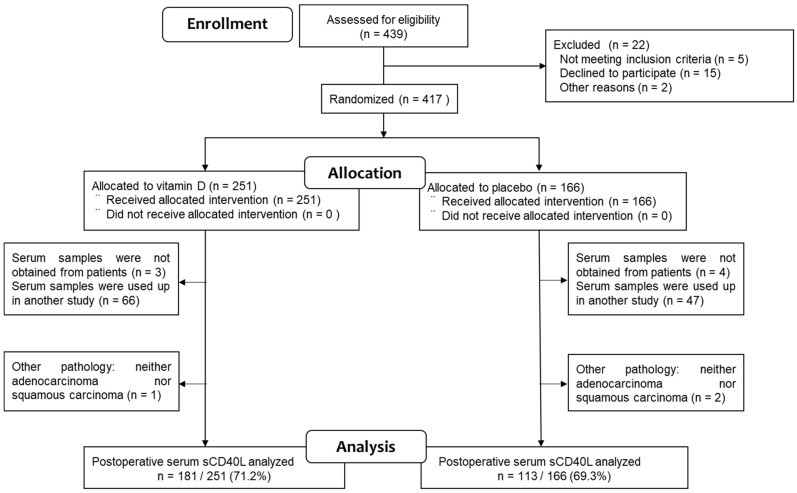
Study flowchart.

A total of 417 patients with digestive tract cancers were initially randomized to receive either vitamin D supplements (n = 251, 60%) or placebo (n = 166, 40%). Importantly, one patient in the vitamin D group was lost to follow-up (drop-out rate, 0.2%). However, serum samples could not be obtained from seven patients (vitamin D group: 3, placebo group: 4). Additionally, 113 serum samples were completely depleted for use in another study (vitamin D group: 66, placebo group: 47), and three patients were excluded due to having pathologies other than adenocarcinoma or squamous cell carcinoma (vitamin D group: 1, placebo group: 2). As a result, the ELISA results for serum sCD40L were available for 294 (70.5%) of the original AMATERASU trial participants (vitamin D group: 181 (72.1%), placebo group: 113 (68.1%)) (Figure 1). The median follow-up period for these 294 patients was 3.3 years (interquartile range (IQR): 1.9–5.2 years).

### 3.2. Characteristics of Patients Randomized into Vitamin D and Placebo Groups

Table 1 provides an overview of the characteristics of patients assigned to the vitamin D and placebo groups. It is important to note that the allocation ratio of 3:2 between the vitamin D and placebo groups was consistently maintained across all variables. Among the 294 participants included in the analysis, approximately 32.7% were women. The median age of the participants was 66 years, with an interquartile range (IQR) of 60–74 years. The median body mass index (BMI) was 21.8 kg/m^2^, falling within an IQR of 19.8–23.8 kg/m^2^.

In terms of cancer distribution, the majority of patients had gastric cancer, accounting for 43.5% of the cohort, followed by colorectal cancer at 46.9%, and esophageal cancer at 9.5%. Cancer stages were categorized as I, II, and III, with 44.2%, 27.2%, and 28.6% of patients, respectively, falling into these stages.

One notable observation was that the vitamin D group appeared to have an older average age compared to the placebo group. Additionally, a higher prevalence of previous cardiovascular disease history was noted in the vitamin D group. However, it is important to emphasize that these differences were considered coincidental due to the randomization process.

### 3.3. Serum sCD40L Levels

**Figure 2:**
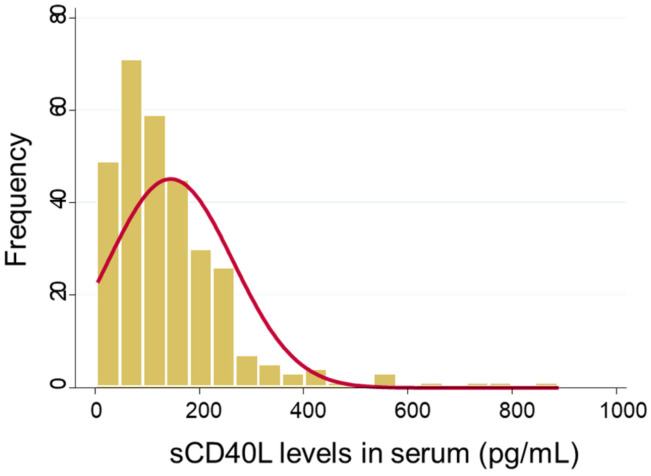
Histogram of serum sCD40L levels.

In our study, we assessed the serum sCD40L levels in a cohort of 294 patients, as illustrated in Figure 2. The median level of sCD40L, presented with the interquartile range (IQR), was found to be 117.3 (67.5–185.9) pg/mL, with a noticeable rightward skew in the distribution. This skew suggests that some patients had notably higher serum sCD40L levels compared to the median, indicating a degree of variability within the study population. The Mann–Whitney test revealed no statistically significant differences in serum sCD40L levels between the vitamin D and placebo groups, with a *p*-value of 0.91.

### 3.4. Characteristics of Patients Stratified into Tertiles According to Serum sCD40L Levels

The results of the detailed examination of the characteristics of our patient cohort, with a focus on their stratification into tertiles based on serum sCD40L levels, are outlined in Table 2.

Of note, patients within the highest tertile of serum sCD40L levels tended to be younger in comparison to their counterparts in the other tertiles (*p* = 0.03). Furthermore, when considering body mass index (BMI), patients in the highest tertile exhibited higher BMIs compared to those in the lowest tertile (*p* = 0.04).

The examination of various other covariates, such as participation in vitamin D intervention, 25(OH)D levels, sex distribution, history of other cancers, comorbidities, pathological characteristics, and the use of adjuvant chemotherapy, revealed that these covariates did not exhibit statistically significant differences among the tertiles.

### 3.5. Survival Analyses of Patients Stratified According to Tertiles of Serum sCD40L

We performed quantitative survival analyses to assess the influence of serum sCD40L levels on relapse or death in the study participants, with a particular focus on patients with CRC.

**Figure 3:**
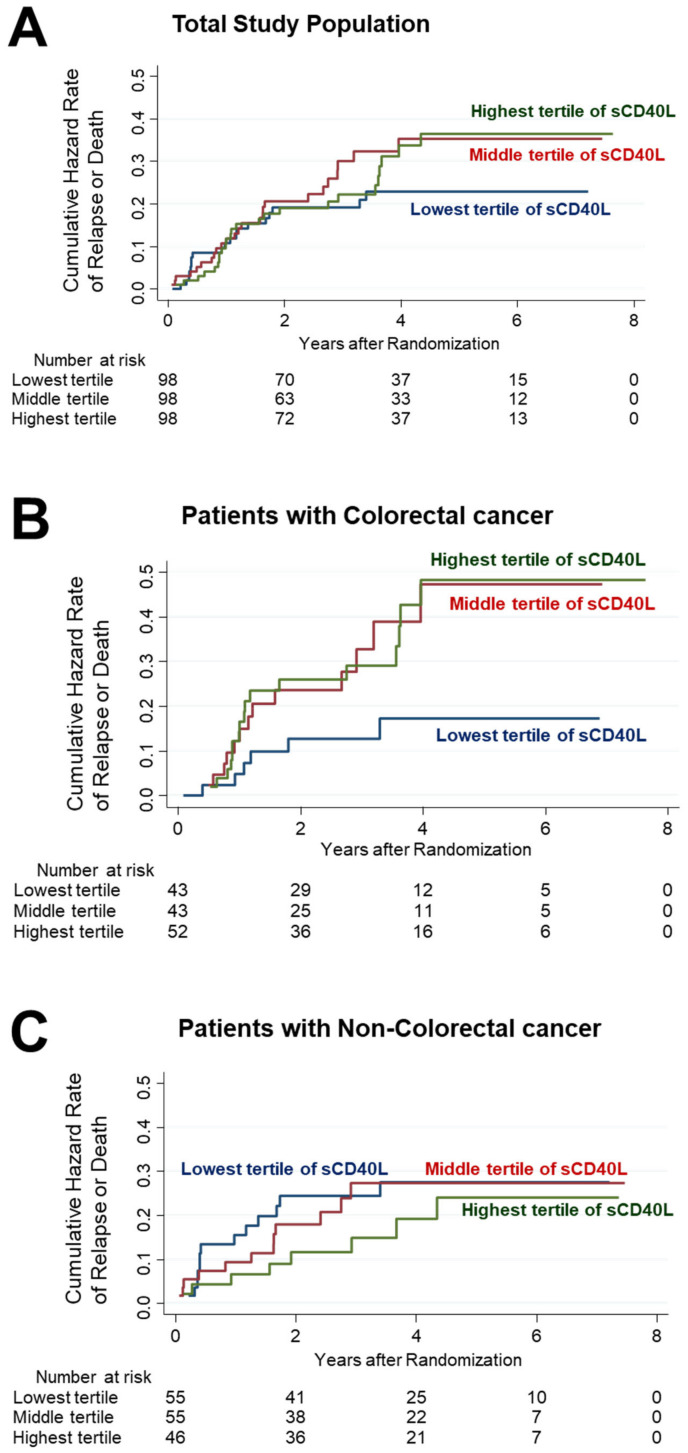
Cumulative hazard curves for relapse or death. Total cohort (**A**), patients with CRC (**B**), and non-CRC patients (**C**).

The analysis of the entire study population revealed 19 events of relapse or death, representing 19.4% of the cohort. Notably, the examination of 5-year RFS rates across tertiles of serum sCD40L levels revealed the following percentages: 79.4% in the lowest sCD40L tertile, 70.1% in the middle tertile, and 69.2% in the highest tertile. However, it is essential to acknowledge that these differences in RFS were not statistically significant within the total study population, irrespective of rigorous adjustment for important covariates such as age, sex, BMI, 25(OH)D levels, vitamin D supplementation or placebo administration, cancer stage, and treatment with adjuvant chemotherapy (Figure 3A).

In contrast, when the focus was narrowed to patients with CRC, the relapse or death rates observed across the sCD40L tertiles were six patients (14.0%) in the lowest sCD40L tertile, thirteen patients (30.2%) in the middle tertile, and seventeen patients (32.7%) in the highest tertile. The 5-year RFS rates among CRC patients exhibited substantial differences, at 83.8% in the lowest sCD40L tertile, 61.6% in the middle tertile, and 61.2% in the highest tertile. Most notably, the Cox proportional hazard analysis, adjusting for the same comprehensive set of variables mentioned above, revealed that patients in the lowest sCD40L level tertile had a significantly better prognosis compared to those in the highest tertile, with a HR of 0.30 and a 95% CI of 0.11–0.80 (*p* = 0.016) (Figure 3B).

However, such differences in RFS were not observed when comparing patients with gastric and esophageal cancers to those with CRC (Figure 3C).

### 3.6. Effect of Vitamin D Supplementation in the Subgroup of CRC Patients in the Highest sCD40L Tertile

The number of patients with CRC was 43 (43.9%) in the lowest, 43 (43.9%) in the middle, and 52 (53.1%) in the highest sCD40L tertile. Although not statistically significant, there was a trend towards a greater number of patients with CRC in the highest tertile. Additionally, although also not statistically significant, the median [IQR] sCD40L level in CRC patients was 126.3 [72.5–200.0] pg/mL, which was higher as compared to non-CRC individuals (115.6 [62.5–185.3] pg/mL). This suggests that CRC tended to be more prevalent in the highest tertile of sCD40L levels.

**Figure 4:**
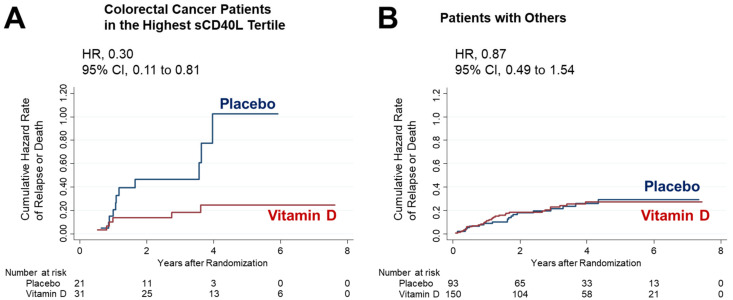
Effect of vitamin D supplementation in the subgroup of patients with colorectal cancer stratified into the highest tertile of sCD40L (**A**), and patients with others, i.e., CRC patients in the middle and lowest tertiles, those with esophageal cancer, and those with gastric cancer (**B**).

Among the 52 patients with CRC and in the highest sCD40L tertile, relapse and death events occurred in 6 of 31 patients (19.4%) in the vitamin D supplementation group and 11 of 21 patients (52.4%) in the placebo group. Within this subgroup, the 5-year RFS in the vitamin D group was notably higher at 77.9%, compared to 33.2% in the placebo group, with a HR of 0.30 and a 95% CI of 0.11–0.81 (*p* = 0.018) (Figure 4A). Importantly, this difference remained significant even after adjusting for age and a history of cardiovascular disease (HR, 0.30; 95% CI, 0.11–0.82; *p* = 0.019).

However, it is essential to highlight that no significant differences were observed between the vitamin D and placebo groups among patients classified in the “others” category, which included patients with CRC not in the highest tertile, those with esophageal cancer, and those with gastric cancer (Figure 4B).

Of note, our analysis indicated a significant interaction between vitamin D supplementation, CRC subgroup, and high sCD40L levels (*P_interaction_* = 0.04).

## 4. Discussion

In this post hoc analysis of the AMATERASU trial [10], we uncovered a significant difference between patients with CRC as compared to the total study population of digestive tract cancers, as well as those with gastric and esophageal cancer. Specifically, we observed that the lowest tertile of sCD40L levels exhibited a substantially reduced risk of relapse or mortality, amounting to less than one-third of the risk observed in the middle and highest sCD40L tertiles. This risk reduction persisted even after adjustment for various factors, including age, sex, BMI, 25(OH)D levels, vitamin D supplementation or placebo administration, cancer stage, and adjuvant chemotherapy. These findings underscore the potential of reduced sCD40L levels as a superior prognostic biomarker specifically in CRC patients.

While sCD40L has been the subject of investigation in various medical contexts, including cognitive aging [45], septic shock in the intensive care unit [46], gestational diabetes mellitus in pregnant women [47], and systemic atherosclerosis [48], its role in the realm of cancer remains relatively uncharted. Hence, the insights gained from our study might hold substantial significance within the field of clinical oncology. Notably, our findings align with prior research indicating higher sCD40L levels in the presence of distant metastases in CRC patients [48], potentially worsening the prognosis of these patients.

Moreover, our discussion regarding the effects of vitamin D supplementation in specific subgroups highlights the potential of vitamin D in improving the prognosis of CRC patients with elevated sCD40L levels. Additionally, compelling literature evidence suggests that sCD40L might contribute to the immunosuppression observed in cancer patients. This might occur through various mechanisms, including sCD40L-enriched myeloid-derived suppressor cells, an additive inhibitory effect on T-cell proliferation, greater sCD40L-induced enrichment of PD-1 expressing T cells, and inhibition of IL-12 production from monocytes [41]. In a previous report, we demonstrated that vitamin D supplementation significantly reduced the risk of relapse or death to approximately one-third in patients with the highest quintile of serum soluble PD-L1, potentially by suppressing serum levels of soluble PD-L1 [11]. Consequently, we hypothesize that elevated sCD40L in the serum of CRC patients might suppress anti-tumor immunity, explaining why CRC patients in the lowest sCD40L tertile exhibited better RFS compared to those in the middle and highest tertiles. Additionally, it is plausible that vitamin D supplementation can mitigate this suppression of anti-tumor immunity induced by sCD40L.

### Study Limitations

Despite these intriguing findings, it is essential to acknowledge the limitations of our study:

1. Sample size: Our analysis included a relatively small number of CRC patients in the highest sCD40L tertile subgroup, as well as in the esophageal cancer group. This limitation might increase the risk of type II errors.
2. Timing of measurements: Serum sCD40L levels were exclusively measured postoperatively, omitting preoperative and post-supplement-initiation measurements. A comprehensive evaluation of whether vitamin D supplementation genuinely reduces sCD40L levels requires a comparison of levels before and after the initiation of vitamin D supplementation and placebo.
3. Exploratory nature: It is crucial to recognize that our study involved exploratory analyses, which were not predefined in the original AMATERASU trial protocol. Consequently, a degree of caution is warranted when interpreting these findings.
4. Subgroup analyses: Subgroup analyses based on tertiles might heighten the probability of type I errors due to multiple comparisons.
5. Population specificity: The AMATERASU trial predominantly featured a Japanese population with specific cancer subtypes. Given the variation in cancer types and genetics across populations, generalizing our study’s outcomes to different populations should be undertaken with caution.

## 5. Conclusions

Overall, our findings underscore the importance of serum sCD40L levels as a prognostic marker in CRC, and suggest that vitamin D supplementation might hold promise in improving outcomes in CRC patients with elevated sCD40L levels. However, it is essential to acknowledge the limitations of our study, including its exploratory nature, sample size constraints, and the specific population under investigation. Further research with larger cohorts and diverse populations is warranted to validate and expand upon these findings, potentially paving the way for personalized treatment strategies in digestive tract cancers.

## Figures and Tables

**Table 1:** Characteristics of patients randomized into vitamin D and placebo groups.

	Vitamin D n = 181	Placebo n = 113
25(OH)D (ng/mL), median IQR (25–75%)	21 (16–27)	21 (14–27)
Sex, n (%)		
Male	127 (70.2)	71 (62.8)
Female	54 (29.8)	42 (37.2)
Age (years), median IQR (25–75%)	67 (61–75)	64 (58–71)
Body mass index (kg/m^2^), median IQR (25–75%)	21.7 (19.6–24.0)	22.1 (20.1–23.6)
History of other cancers, n (%)	6 (5.3)	6 (3.3)
Comorbidities, n (%)		
Hypertension	72 (39.8)	42 (37.2)
Diabetes mellitus	33 (18.2)	15 (13.3)
Endocrine diseases	22 (12.2)	10 (8.9)
Cardiovascular diseases	15 (8.3)	7 (6.2)
Chronic kidney diseases	3 (1.7)	0 (0.0)
Asthma	3 (1.7)	0 (0.0)
Orthopedic diseases	0 (0.0)	1 (0.9)
Site of cancer, n (%)		
Esophagus	16 (8.8)	12 (10.6)
Stomach	76 (42.0)	52 (46.0)
Colorectal	89 (49.2)	49 (43.4)
Stage, n (%)		
I	82 (45.3)	48 (42.5)
II	46 (25.4)	34 (30.1)
III	53 (29.3)	31 (27.4)
Pathology		
Adenocarcinoma, n (%)	165 (91.2)	102 (90.3)
Squamous cell carcinoma, n (%)	16 (8.8)	11 (9.7)
Adjuvant chemotherapy, n (%)	63 (34.8)	40 (35.4)

**Table 2:** Characteristics of patients stratified into tertiles of serum sCD40L levels.

	Total, n = 294	Lowest Tertile, n = 98	Middle Tertile, n = 98	Highest Tertile, n = 98	*p*-Value
sCD40L (pg/mL), median (IQR)	117 (67–186)	49 (32–67)	117 (98–138)	229 (186–276)	
Vitamin D supplementation, n (%)	181 (61.6)	59 (60.2)	65 (66.3)	57 (58.2)	0.47 ^c^
25(OH)D (ng/mL), median (IQR)	21 (16–27)	20 (16–27)	22 (16–29)	20.5 (15–27)	0.32 ^b^
Female, n (%)	96 (32.7)	32 (32.7)	30 (30.6)	34 (34.7)	0.83 ^c^
Age (years), median (IQR)	66 (60–74)	67 (61–74)	67 (62–74)	64 (57–72)	0.03 ^b^
BMI (kg/m^2^), median (IQR)	21.8 (19.8–23.8)	21.3 (19.5–23.3)	21.8 (20.0–23.6)	22.2 (20.2–24.5)	0.04 ^b^
History of other cancers, n (%)	12 (4.1)	4 (4.1)	3 (3.1)	5 (5.1)	0.77 ^c^
Comorbidities, n (%)					
Hypertension	114 (38.8)	38 (38.8)	36 (36.7)	40 (40.8)	0.84 ^c^
Diabetes mellitus	48 (16.3)	14 (14.3)	18 (18.4)	16 (16.3)	0.74 ^c^
Endocrine diseases	32 (10.9)	10 (10.2)	8 (8.2)	14 (14.3)	0.38 ^c^
Cardiovascular diseases	22 (7.5)	7 (7.1)	5 (5.1)	10 (10.2)	0.39 ^c^
Chronic kidney diseases	3 (1.0)	1 (1.0)	1 (1.0)	1 (1.0)	1.00 ^c^
Asthma	3 (1.0)	1 (1.0)	1 (1.0)	1 (1.0)	1.00 ^c^
Orthopedic diseases	1 (0.3)	1 (1.0)	0 (0.0)	0 (0.0)	0.37 ^c^
Site of cancer, n (%) ^a^					0.22 ^c^
Esophagus	28 (9.5)	14 (14.3)	7 (7.1)	7 (7.1)	
Stomach	128 (43.5)	41 (41.8)	48 (49.0)	39 (39.8)	
Colorectal	138 (46.9)	43 (43.9)	43 (43.9)	52 (53.1)	
Stage, n (%) ^a^					0.24 ^c^
I	130 (44.2)	36 (36.7)	49 (50.0)	45 (45.9)	
II	80 (27.2)	31 (31.6)	27 (27.6)	22 (22.5)	
III	84 (28.6)	31 (31.6)	22 (22.5)	31 (31.6)	
Pathology, n (%)					0.10 ^c^
Adenocarcinoma	267 (90.8)	84 (85.7)	92 (93.9)	91 (92.9)	
Squamous cell carcinoma	27 (9.2)	14 (14.3)	6 (6.1)	7 (7.1)	
Adjuvant chemotherapy, n (%)	103 (35.0)	43 (43.9)	29 (29.6)	31 (31.6)	0.08 ^c^

^a^ Percentages might not add up to 100% because of rounding. ^b^ *p*-value was calculated using the Kruskal–Wallis equality-of-populations rank test. ^c^ *p*-value was calculated using the chi-square test.

## Data Availability

The principal investigator (urashima@jikei.ac.jp) had full access to all the study data and takes responsibility for the integrity of the data and accuracy of the data analysis. The data will be available upon reasonable request to the principal investigator.
